# Delta MEWS combined with SpO_2_ for identifying MEWS-based high-alert status at unplanned transfer to the emergency resuscitation room: a multicenter retrospective study

**DOI:** 10.3389/fmed.2026.1791335

**Published:** 2026-06-03

**Authors:** Hongquan Fan, Quan Yuan, Fengjiang Qu, Zhe Chu, Lupeng Cui

**Affiliations:** 1 Emergency Department, The First Hospital of Jilin University, Changchun, China; 2 Academic Affairs Office, The First Hospital of Jilin University, Changchun, China

**Keywords:** Delta MEWS, emergency observation, emergency resuscitation room, nomogram, physiologic deterioration, SpO_2_

## Abstract

**Objective:**

To develop and validate a practical model to identify MEWS-based high-alert physiologic status at the time of unplanned transfer to the emergency resuscitation room (UTER) among emergency patients under observation.

**Methods:**

This multicenter retrospective study used complete-case data from three hospitals in China. The training cohort comprised 599 UTER patients from the First Hospital of Jilin University (January 1 – December 31, 2023). Temporal internal validation included 145 UTER patients from the same hospital (January 1 – March 31, 2024). Geographic external validation included 231 UTER patients from Hainan Provincial People’s Hospital and the Second Hospital of Shanxi Medical University (December 1, 2022 – March 1, 2023). M1 was the MEWS within 1 h after entry to the emergency observation room; M2 was the MEWS at UTER; Delta MEWS = M2 – M1. The modeled endpoint was MEWS-based high-alert status at UTER, defined as M2 ≥ 6. Logistic regression, nomogram construction, ROC analysis, calibration analysis, and decision-curve analysis were used. The positive class for precision-recall analysis was prespecified as MEWS-based high-alert status at UTER (M2 ≥ 6).

**Results:**

In the training cohort, the optimal training-set cutoff for Delta MEWS was 3.5, corresponding to a sensitivity of 0.837 and a specificity of 0.924. In multivariable analysis, SpO_2_ at UTER (OR 0.97, 95% CI 0.94–1.00; *p* = 0.027) and Delta MEWS ≥ 3.5 (OR 53.26, 95% CI 29.51–96.11; *p* < 0.001) were independently associated with high-alert status. The model achieved an AUROC of 0.890 (95% CI 0.853–0.927) in the training cohort, 0.957 (95% CI 0.917–0.997) in temporal internal validation, and 0.934 (95% CI 0.871–0.997) in geographic external validation. The corresponding AUCPR values were 0.729 (95% CI 0.673–0.781), 0.921 (95% CI 0.872–0.991) and 0.618 (95% CI 0.542–0.723), compared with cohort-specific positive-class fractions of 23.5, 33.1, and 6.5%, respectively.

**Conclusion:**

Delta MEWS combined with SpO_2_ provided bedside stratification of MEWS-based high-alert status at UTER. The nomogram may assist clinicians in recognizing severe physiologic worsening during transfer. Reporting precision-recall performance together with class balance clarifies model discrimination across cohorts with different prevalences.

## 1 Introduction

In high-volume emergency observation areas, some patients who initially appear relatively stable can deteriorate between intermittent assessments. Because these units often manage large numbers of patients, rapid turnover, mixed disease spectra, and limited continuous monitoring resources, subtle physiologic worsening may be overlooked until urgent escalation is required. Vital-sign surveillance is therefore central to early detection of deterioration, timely intervention, and patient safety in emergency nursing and acute care practice (1, 2).

Modified Early Warning Score (MEWS) is a standardized bedside score based on routinely collected physiologic variables and has been widely used to identify clinical deterioration in hospitalized and emergency patients. Since its introduction by Subbe et al., MEWS has been applied across wards, emergency departments, and prehospital settings (3). Prior studies have shown that vital signs often begin to change before major clinical deterioration becomes clinically obvious (4–6). Accurate recording of respiratory and other physiologic parameters is therefore important when evaluating unstable patients, and elevated early warning scores have been associated with worse short-term outcomes (7, 8).

Monitoring physiologic parameters is especially important for patients managed in emergency observation areas. MEWS and SpO_2_ are among the most readily available bedside indicators in this setting, and previous studies have linked early warning scores with hospital admission, serious adverse events, mortality, and other markers of acute deterioration (9–12). However, most previous studies have focused on outcomes such as admission, ICU transfer, death, or return visits rather than physiologic stratification at the moment emergency patients under observation undergoes unplanned transfer to the emergency resuscitation room (UTER).

Accordingly, the present study aimed to develop and validate a practical bedside model based on Delta MEWS and SpO_2_ to stratify MEWS-based high-alert physiologic status at the time of UTER among emergency observation patients who had already undergone UTER. The study therefore focuses on physiologic stratification at the transfer event itself rather than prediction of future UTER occurrence in the broader observation-room population.

## 2 Materials and methods

### 2.1 Study design, setting, and cohort timeline

This was a retrospective multicenter observational study involving the First Hospital of Jilin University, Hainan Provincial People’s Hospital, and the Second Hospital of Shanxi Medical University. The training cohort included UTER patients from the First Hospital of Jilin University between January 1 and December 31, 2023. The temporal internal validation cohort included UTER patients from the same hospital between January 1 and March 31, 2024. The geographic external validation cohort included UTER patients from Hainan Provincial People’s Hospital and the Second Hospital of Shanxi Medical University between December 1, 2022 and March 1, 2023.

**Figure 1:**
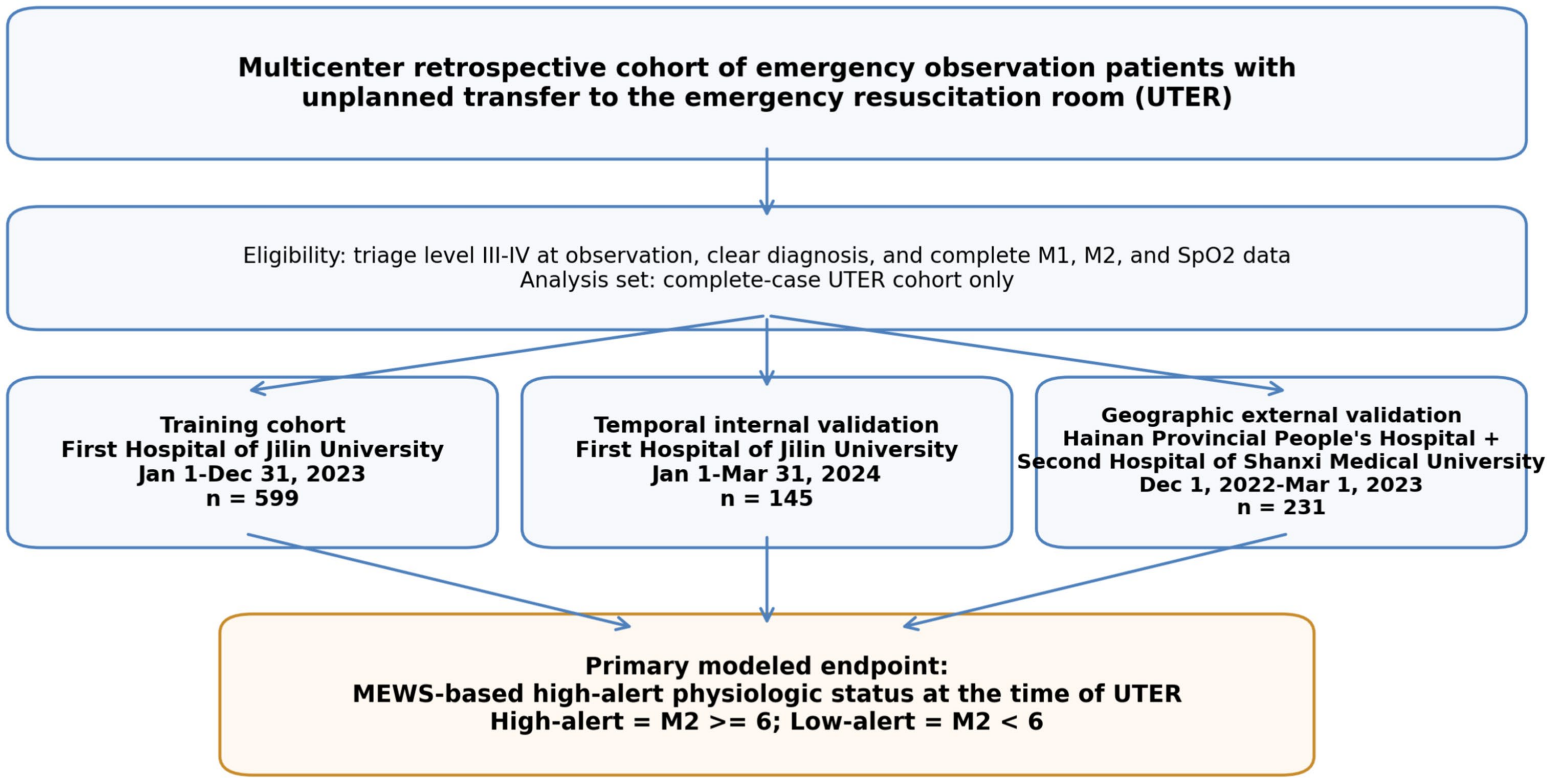
Study design, cohort construction, and modeled endpoint.

This design allowed temporal validation within the same center and geographic validation across independent hospitals. The study flow and endpoint definition are summarized in Figure 1.

### 2.2 Study participants and endpoint

Eligible patients met all of the following criteria: (1) triage level III or IV at observation; (2) a clear primary diagnosis and observation according to the emergency physician’s order; (3) unplanned transfer from the emergency observation area to the emergency resuscitation room because of clinical worsening. In accordance with the Chinese expert consensus on emergency triage, levels III and IV correspond to urgent and sub-urgent patients who generally require treatment and observation rather than immediate resuscitation (13). Patients with unclear diagnoses or missing key data were excluded.

Because only patients who had already undergone UTER were included, the modeled outcome was not a pre-transfer prediction of whether observation-room patients would experience UTER. Instead, the primary endpoint was MEWS-based high-alert physiologic status at the time of transfer, defined as M2 ≥ 6 versus M2 < 6. This threshold was chosen in accordance with published literature that has used MEWS ≥6 to identify patients at high risk of further deterioration (14).

The MEWS scores and vital-sign variables were extracted from a structured electronic vital-sign recording system in the emergency department. During clinical screening, missingness was observed to be very uncommon and was estimated to be <1%. However, the currently available retrospective screening logs did not preserve a reliable denominator for all incomplete records, so the exact missing-data proportion could not be reconstructed without risking an inaccurate estimate. Therefore, complete-case analysis was used. This issue and the related possibility of limited selection bias are acknowledged in the Discussion.

### 2.3 Variables and measurements

The collected variables included age, sex, primary diagnostic category, MEWS within 1 h after entry to the emergency observation room (M1), MEWS at UTER (M2), and SpO_2_ at UTER. Delta MEWS was defined as M2 – M1. The MEWS system comprised respiratory rate, systolic blood pressure, heart rate, temperature, and level of consciousness. Because respiratory abnormalities are a particularly important early sign of deterioration, careful recording of respiratory parameters remains clinically important when using bedside warning scores (7).

Based on the emergency physician’s primary diagnosis, diseases were classified into nine groups: urinary system diseases; endocrine system diseases; digestive system diseases; respiratory system diseases; cardiovascular system diseases; neurological disorders; poisoning, trauma, and allergies; hematologic diseases; and ENT/ocular/neck disorders.

Candidate variables for multivariable modeling were selected according to clinical availability at the bedside and univariable screening. Delta MEWS was initially evaluated as a dynamic marker and was then dichotomized at the training-set cutoff of 3.5 to improve bedside usability and interpretability. Although dichotomization may lead to some loss of information, a simple cutoff was considered clinically practical for emergency nurses and physicians during shift work (Table 1).

**Table 1:** Assignment of values to the indicators of the MEWS score.

Variables	MEWS score						
	3	2	1	0	1	2	3
Temperature (C)		≤35.0	35.0–36.1	36.1–38.0	38.1–38.5	>38.5	
Respiratory rate (breaths/min)		≤8		9–14	15–20	21–29	>29
Heart rate (beats/min)		≤40	41–45	51–100	101–110	111–130	>130
SBP (mmHg)	≤70	71–80	81–100	101–199		>200	
AVPU score				A	V	P	U

AVPU = Alert, Voice, Pain, Unresponsive.

### 2.4 Statistical analysis

R (version 4.2.1) was used for statistical analysis. Continuous variables were assessed for distributional characteristics and summarized as mean ± standard deviation or median (interquartile range), as appropriate. Group comparisons used the independent-sample *t* test, Mann–Whitney U test, or Chi-square test.

Univariable logistic regression was first performed for candidate variables. Variables with clinical relevance and/or a univariable *p* value < 0.10 were considered for multivariable analysis. The final model retained the bedside-available variables that remained independently associated with the endpoint. Collinearity was assessed using the variance inflation factor (VIF). Given the very low estimated missingness (<1%) and the inability to reconstruct exact missingness retrospectively, no imputation was performed and complete-case analysis was applied.

The optimal cutoff for Delta MEWS was derived from the training cohort using ROC analysis and the maximum Youden index. Model performance was evaluated using ROC curves, calibration curves, Hosmer–Lemeshow testing, decision-curve analysis, and clinical impact curves in the training, temporal internal validation, and geographic external validation cohorts.

In addition, precision-recall performance was assessed because precision-recall summaries are informative when class imbalance differs across cohorts. The positive class was defined *a priori* as MEWS-based high-alert status at UTER (M2 ≥ 6; y = 1), whereas M2 < 6 was the negative class (y = 0). For each cohort, patient-level predicted probabilities from the final logistic model were used to generate precision-recall curves. The primary summary measure was the area under the precision-recall curve (AUCPR), computed in R using the PRROC package (version 1.4; function `pr.curve`) with the `auc.integral` (piecewise non-linear) integration rule. To facilitate interpretation, AUCPR was reported together with the cohort-specific positive-class fraction (baseline precision). Ninety-five percent confidence intervals for AUCPR were estimated by nonparametric bootstrap with 2,000 patient-level resamples.

For nomogram interpretation, a risk probability of 0.5 was used as a reference threshold. Bedside thresholds should be adapted to local clinical settings and informed by decision-curve analysis. A two-sided *p* < 0.05 was considered statistically significant.

### 2.5 Ethics

This study was approved by the Ethics Committee of the First Hospital of Jilin University (2023 I KS I 136). The external validation cohorts were also approved by the Ethics Committees of Hainan Provincial People’s Hospital and the Second Hospital of Shanxi Medical University. All methods were performed in accordance with relevant guidelines and regulations.

Because this was a retrospective study based on routinely collected clinical data and did not report identifiable patient information, the requirement for written informed consent was waived by the ethics committee.

## 3 Results

According to the inclusion and exclusion criteria, 599 UTER patients from the emergency department of the First Hospital of Jilin University between January 1 and December 31, 2023 were enrolled as the training cohort. Another 145 UTER patients from the same hospital between January 1 and March 31, 2024 were enrolled as the temporal internal validation cohort. In addition, 231 UTER patients from Hainan Provincial People’s Hospital and the Second Hospital of Shanxi Medical University between December 1, 2022 and March 1, 2023 were enrolled as the geographic external validation cohort.

### 3.1 Optimal cutoff selection for Delta MEWS

**Figure 2:**
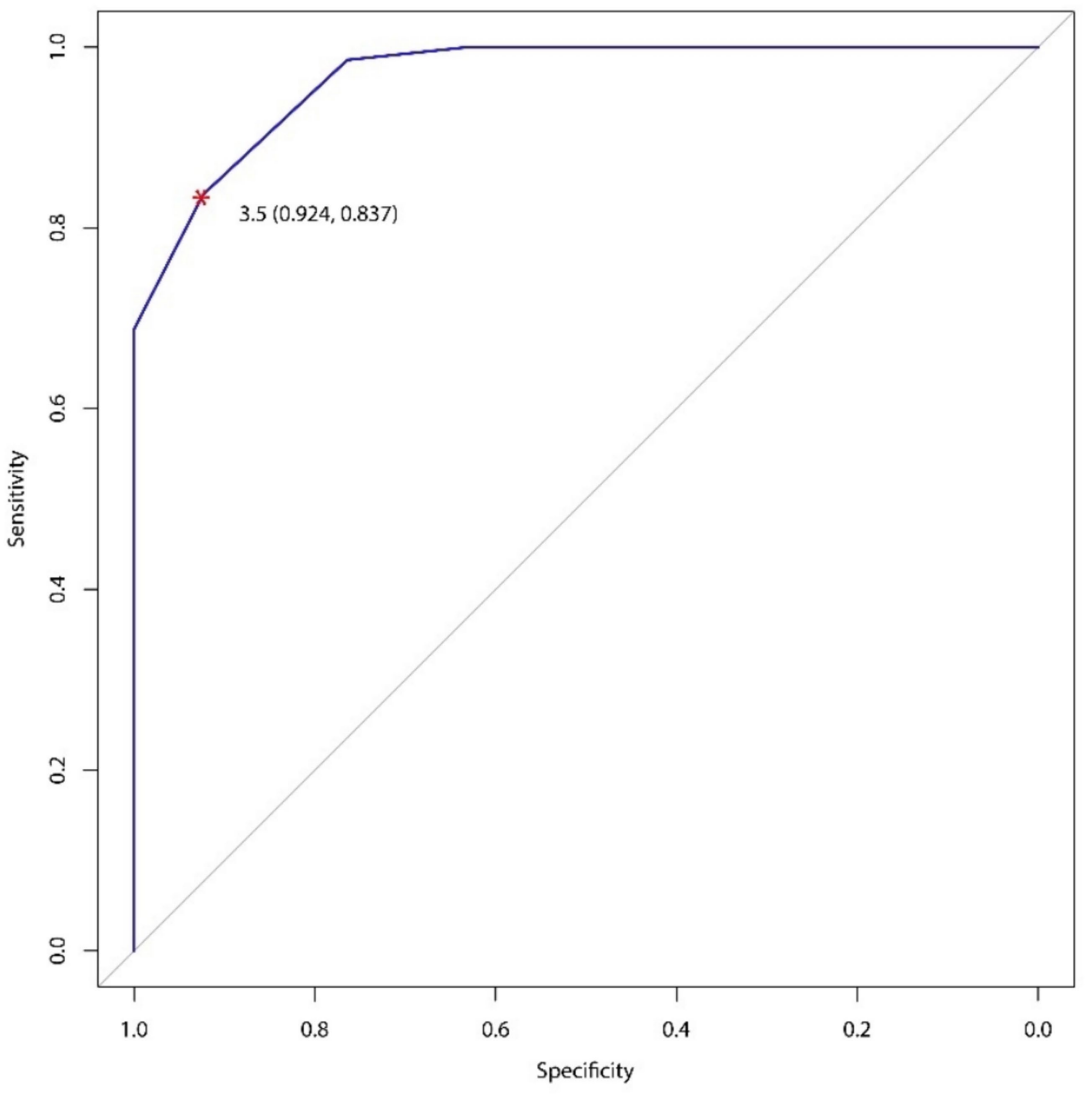
AUROC analysis used to derive the representative cutoff for delta MEWS in the training cohort.

To simplify bedside judgment, a representative training-set cutoff for Delta MEWS was derived. AUROC analysis based on Delta MEWS alone showed that the maximum Youden index was 0.761, corresponding to a sensitivity of 0.837 and a specificity of 0.924. The associated Delta MEWS value was 3.5 (Figure 2).

Clinically, a cutoff of 3.5 indicates that an increase of approximately 4 MEWS points identifies most patients in the high-alert group while maintaining high specificity. Accordingly, Delta MEWS ≥3.5 was categorized as the high-alert-change group and Delta MEWS < 3.5 as the low-alert-change group for nomogram construction and bedside interpretation.

### 3.2 Logistic regression for MEWS-based high-alert status at UTER

In the multivariable model, SpO_2_ at UTER (*p* = 0.027) and Delta MEWS ≥ 3.5 (*p* < 0.001) remained independently associated with MEWS-based high-alert status at transfer. The corresponding slope coefficients for the final model were −0.0305 for SpO_2_ and 3.975 for Delta MEWS ≥ 3.5. Collinearity analysis showed no relevant covariance between SpO_2_ and Delta MEWS (VIF = 1.0011 for both) (Table 2).

**Table 2:** Univariable and multivariable logistic regression for MEWS-based high-alert status at UTER in the training cohort.

Item	Category	Low-alert group (*N* = 458)	High-alert group (*N* = 141)	Univariable OR	*p* value	Multivariable OR	*p* value
Age		59.9 ± 16.5	61.3 ± 15.8	1.01 (0.99–1.02)	0.354		
Gender	Female	186 (40.6%)	43 (30.5%)	Ref	-	Ref	-
	Male	272 (59.4%)	98 (69.5%)	1.56 (1.04–2.33)	0.031	1.62 (0.89–2.95)	0.121
Urinary system diseases	No	454 (99.1%)	140 (99.3%)	Ref	-		
	Yes	4 (0.9%)	1 (0.7%)	0.81 (0.09–7.31)	0.852		
Endocrine system diseases	No	443 (96.7%)	139 (98.6%)	Ref	-		
	Yes	15 (3.3%)	2 (1.4%)	0.42 (0.10–1.88)	0.260		
Digestive system diseases	No	390 (85.2%)	134 (95.0%)	Ref	-	Ref	-
	Yes	68 (14.8%)	7 (5.0%)	0.30 (0.13–0.67)	0.003	0.58 (0.19–1.76)	0.341
Respiratory system diseases	No	318 (69.4%)	109 (77.3%)	Ref	-		
	Yes	140 (30.6%)	32 (22.7%)	0.67 (0.43–1.04)	0.072		
Cardiovascular system diseases	No	434 (94.8%)	134 (95.0%)	Ref	-		
	Yes	24 (5.2%)	7 (5.0%)	0.94 (0.40–2.24)	0.897		
Neurological disorders	No	289 (63.1%)	57 (40.4%)	Ref	-	Ref	-
	Yes	169 (36.9%)	84 (59.6%)	2.52 (1.71–3.71)	<0.001	1.16 (0.62–2.17)	0.638
Poisoning, trauma, allergies	No	438 (95.6%)	138 (97.9%)	Ref	-		
	Yes	20 (4.4%)	3 (2.1%)	0.48 (0.14–1.63)	0.236		
Hematologic diseases	No	442 (96.5%)	136 (96.5%)	Ref	-		
	Yes	16 (3.5%)	5 (3.5%)	1.02 (0.37–2.82)	0.976		
ENT/ocular/neck disorders	No	456 (99.6%)	141 (100%)	Ref	-		
	Yes	2 (0.4%)	0 (0%)	0 (0-Inf)	0.983		
SpO_2_ at UTER		93.9 ± 6.8	85.7 ± 23.7	0.96 (0.94–0.97)	<0.001	0.97 (0.94–1.00)	0.027
Delta MEWS	<3.5	423 (92.4%)	23 (16.3%)				
	≥3.5	35 (7.6%)	118 (83.7%)	62.00 (35.27–109.02)	<0.001	53.26 (29.51–96.11)	<0.001

The final logistic equation was expressed as: Logit(P) = 2.198–0.0305 x SpO_2_ + 3.975 x Delta MEWS_group, where Delta MEWS_group was coded as 1 for Delta MEWS ≥ 3.5 and 0 for Delta MEWS <3.5. Accordingly, *p* = 1/[1 + exp.(−(2.198–0.0305 × SpO_2_ + 3.975 × Delta MEWS_group))].

**Figure 3:**
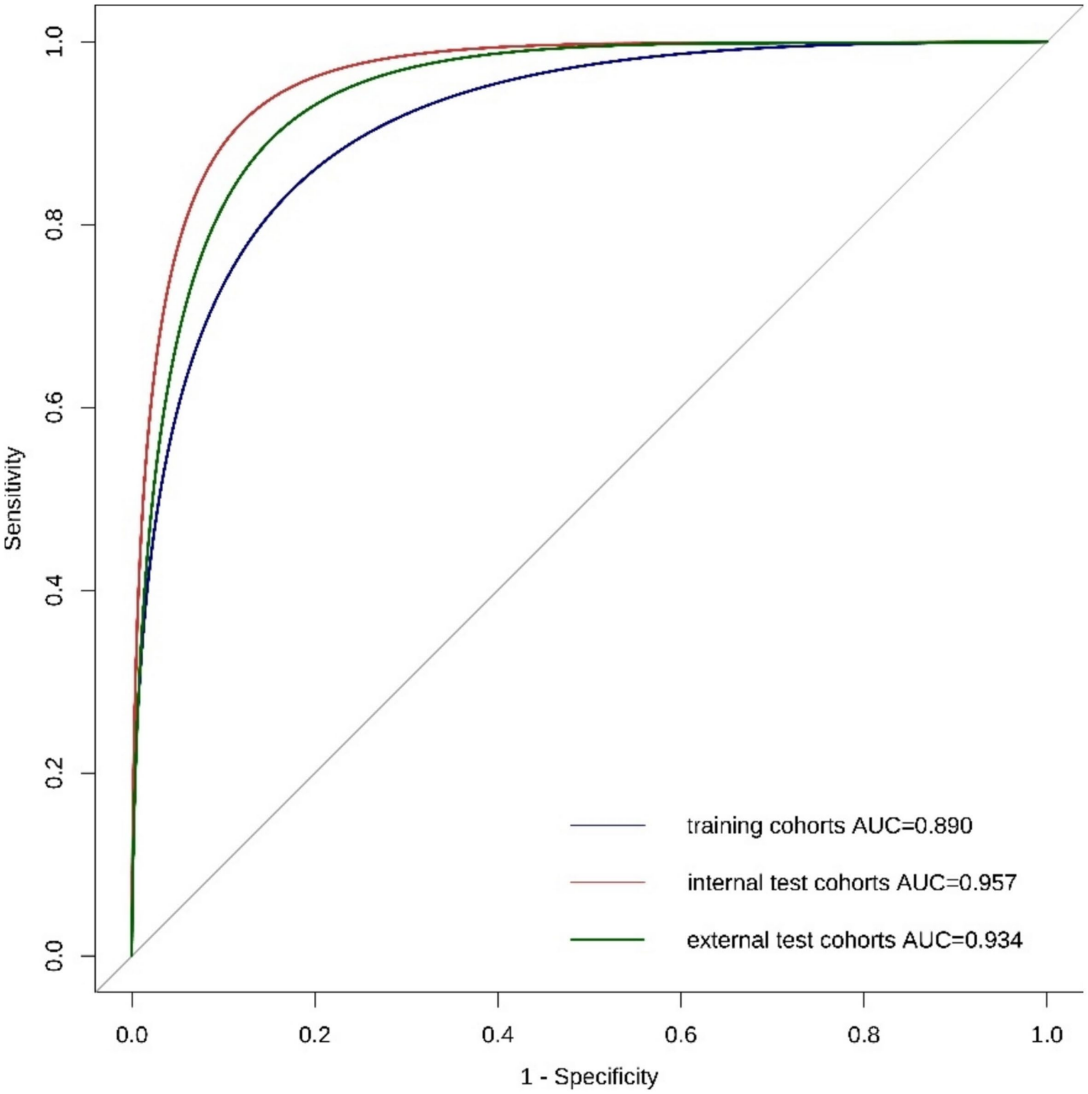
AUROC curves for the training, temporal internal validation, and geographic external validation cohorts.

Because Delta MEWS is derived from MEWS and the endpoint is also defined using M2, the predictor and endpoint are structurally related. The model should therefore be interpreted as a stratification tool for a MEWS-based severity threshold at UTER rather than as a fully independent prognostic model for hard clinical outcomes (Figure 3).

### 3.3 Model discrimination, calibration, and clinical utility

The final model achieved an AUC of 0.890 (95% CI 0.853–0.927) in the training cohort. The corresponding AUCs were 0.957 (95% CI 0.917–0.997) in temporal internal validation and 0.934 (95% CI 0.871–0.997) in geographic external validation, indicating good discrimination across both validation settings (Table 3).

**Table 3:** Discrimination performance and class balance across development and validation cohorts.

Cohort	Positive class fraction	Accuracy	Precision	Sensitivity	Specificity	F1 score	AUROC (95% CI)	AUCPR (95% CI)
Training	0.235	0.903	0.771	0.837	0.924	0.803	0.890 (0.853–0.927)	0.729 (0.673–0.781)
Temporal internal validation	0.331	0.924	0.930	0.833	0.969	0.879	0.957 (0.917–0.997)	0.921 (0.872–0.991)
Geographic external validation	0.065	0.944	0.550	0.733	0.958	0.629	0.934 (0.871–0.997)	0.618 (0.542–0.723)

**Figure 4:**
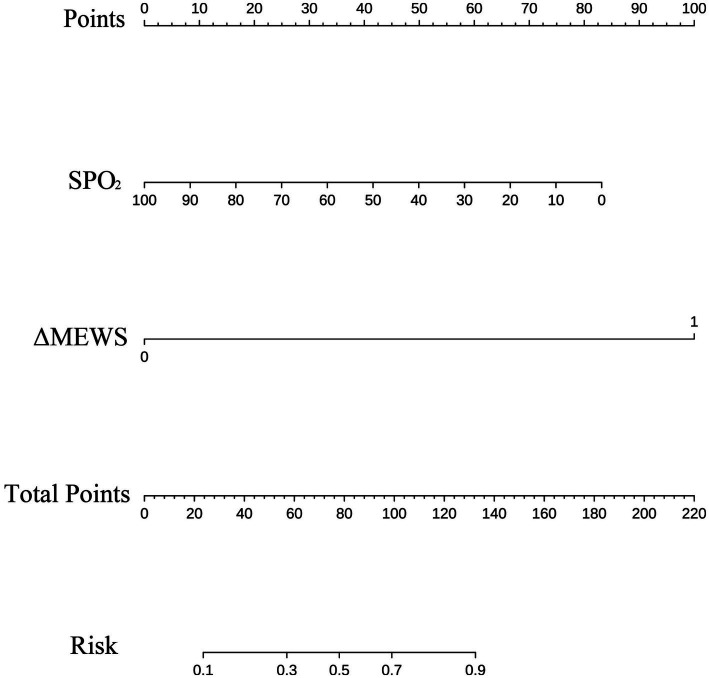
Nomogram for bedside estimation of MEWS-based high-alert status at the time of UTER.

Based on the multivariable model, a nomogram was constructed for rapid bedside use (Figure 4). In the figure, a risk probability of 0.5 is shown as an illustrative reference threshold; however, the decision-curve results indicate that clinically useful thresholds can extend across a broader range.

### 3.4 Model discrimination, calibration, and clinical utility

The final model achieved an AUROC of 0.890 (95% CI 0.853–0.927) in the training cohort. The corresponding AUROC values were 0.957 (95% CI 0.917–0.997) in temporal internal validation and 0.934 (95% CI 0.871–0.997) in geographic external validation, indicating good discrimination across cohorts.

To complement AUROC, precision-recall performance was evaluated against cohort-specific class balance. The AUCPR was 0.729 (95% CI 0.673–0.781) in the training cohort 0.921 (95% CI 0.872–0.991) in the temporal internal validation cohort, and 0.618 (95% CI 0.542–0.723) in the geographic external validation cohort. The corresponding positive-class fractions were 141/599 (23.5%), 48/145 (33.1%), and 15/231 (6.5%), respectively. AUCPR was therefore interpreted relative to the positive-class fraction in each cohort rather than in isolation. In particular, the geographic external cohort had a much lower prevalence, so its AUCPR should be judged against a lower baseline.

Based on the final model, a nomogram was constructed (Figure 4). In the figure, a risk probability of 0.5 is shown as an illustrative reference; however, decision-curve analysis indicates that useful thresholds can extend across a wider range.

The Hosmer–Lemeshow test indicated acceptable calibration in the training cohort (*χ*^2^ = 8.19, *p* = 0.316), temporal internal validation cohort (*χ*^2^ = 5.11, *p* = 0.746), and geographic external validation cohort (*χ*^2^ = 3.08, *p* = 0.687). The decision curve in the training cohort suggested net benefit across a broad threshold range, and the clinical impact curve showed better alignment between predicted and observed high-risk cases at higher thresholds.

**Figure 5:**
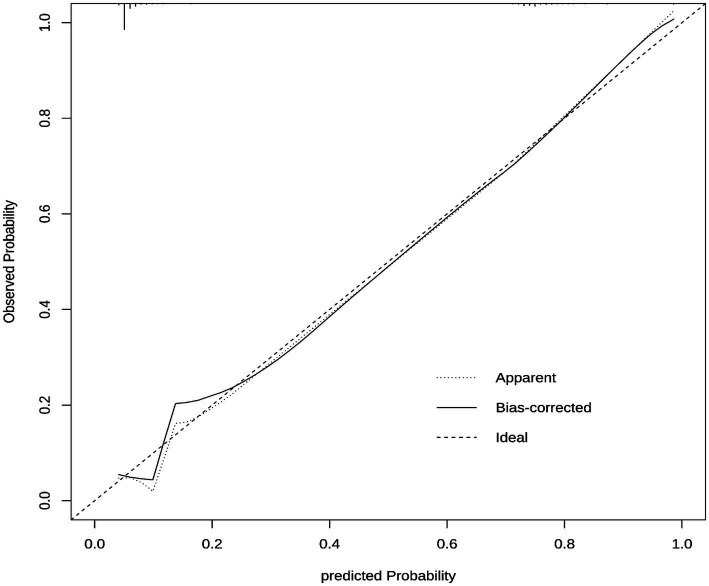
Calibration curve of the model in the training cohort.

**Figure 6:**
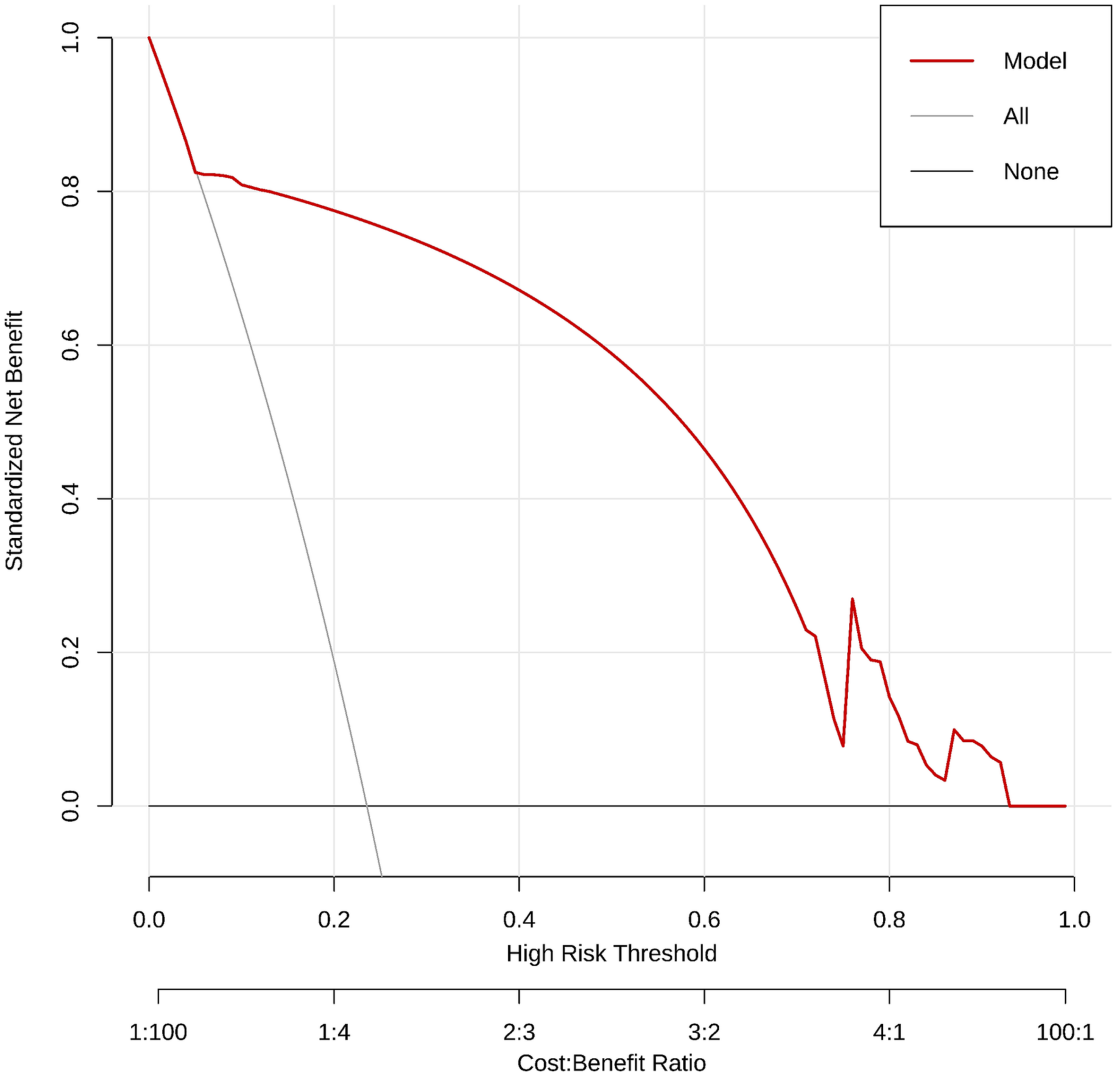
Decision-curve analysis of the model in the training cohort.

**Figure 7:**
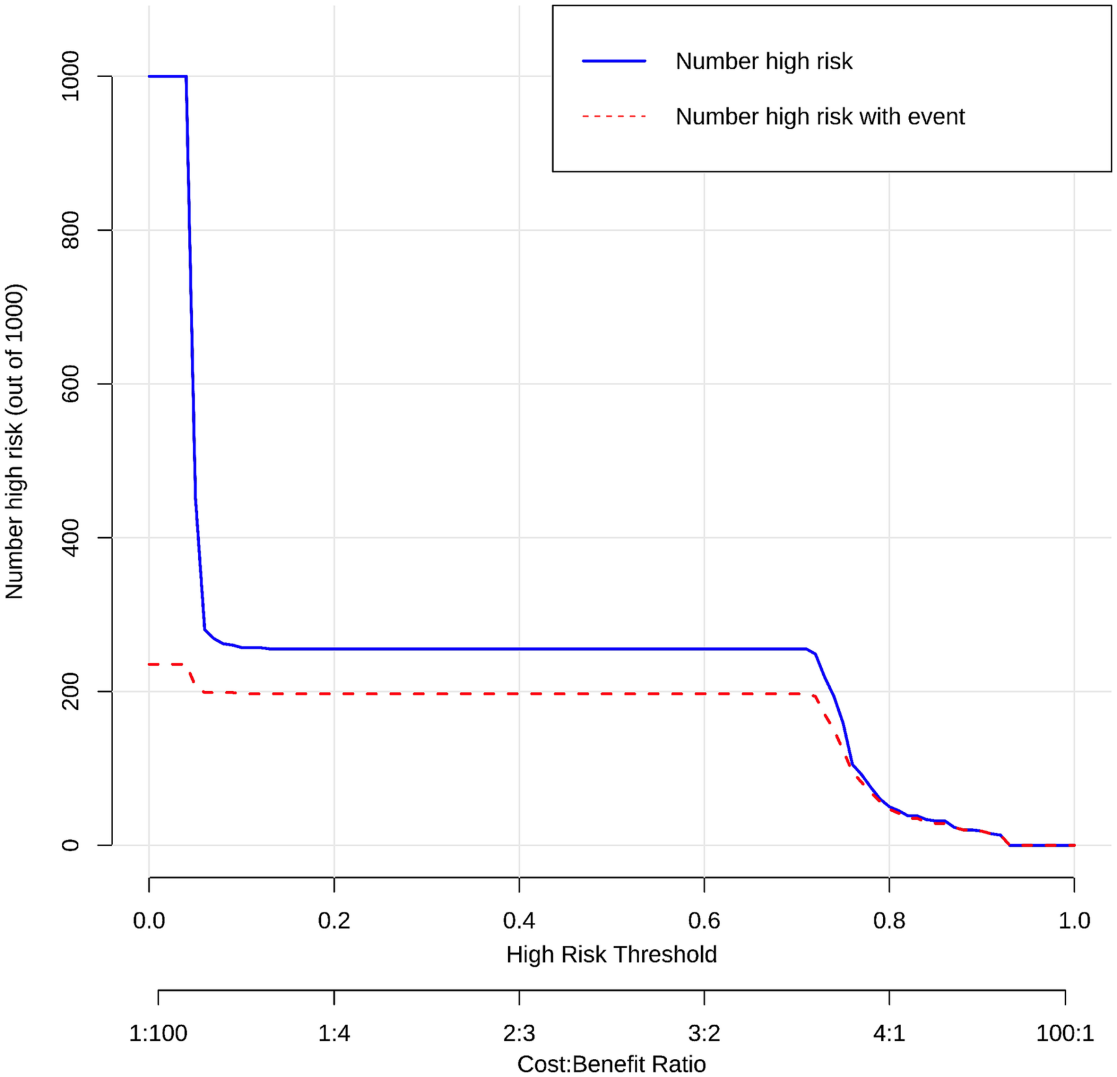
Clinical impact curve of the model in the training cohort.

The Hosmer–Lemeshow test indicated acceptable calibration in the training cohort (Chi-square = 8.19, *p* = 0.316), temporal internal validation cohort (Chi-square = 5.11, *p* = 0.746), and geographic external validation cohort (Chi-square = 3.08, *p* = 0.687). The training-cohort decision curve suggested net clinical benefit across a broad threshold range (approximately 0.05–1.0), and the clinical impact curve showed better alignment between predicted and observed high-risk cases when the threshold exceeded approximately 0.6 (Figures 5–7).

## 4 Discussion

### 4.1 Main findings and correct interpretation of the endpoint

This multicenter retrospective study developed and validated a practical model combining Delta MEWS and SpO_2_ to stratify MEWS-based high-alert physiologic status at the time of UTER among emergency observation patients who had already undergone UTER. This topic is clinically relevant because observation areas in emergency departments often accommodate many patients with varied diseases, rapid turnover, and limited continuous monitoring; under these conditions, latent deterioration may be missed until the patient requires urgent transfer (15, 16). A bedside tool that highlights high-alert physiologic status at the moment of transfer may therefore help clinicians prioritize escalation, monitoring intensity, and documentation.

Accordingly, the present model is best interpreted as a bedside stratification tool for MEWS-based high-alert physiologic status at UTER rather than as a pre-transfer prediction model for the broader emergency observation-room population. The addition of precision-recall analysis strengthens the interpretation of discrimination. Because precision-recall summaries depend on class balance, AUCPR should be interpreted together with the positive-class fraction in each cohort rather than against an absolute cutoff. This is clinically relevant here because the proportion of high-alert cases differed across our cohorts, and AUCPR therefore provides complementary information to AUROC about the concentration of true high-alert cases among those predicted high risk.

### 4.2 Clinical meaning of the Delta MEWS cutoff and dynamic physiologic assessment

A Delta MEWS cutoff of 3.5 was selected from the training cohort because it balanced sensitivity (83.7%) and specificity (92.4%). In practice, this means that an increase of approximately 4 MEWS points should prompt strong concern that the patient has already entered a substantially higher-alert physiologic state at transfer. This threshold may help bedside staff make faster judgments during busy shifts, but it should be interpreted together with the patient’s overall clinical picture rather than used in isolation.

The dynamic aspect of the model is also noteworthy. Acute deterioration is often a pAUROCess rather than a single point event, and prior studies have shown that abnormal vital signs may appear hours before catastrophic outcomes such as unexpected ICU transfer or cardiopulmonary arrest (17–19). Early warning scores used in emergency and acute-care settings have shown value for identifying deterioration, while oxygenation-related markers such as the SpO_2_/FiO_2_ ratio may also signal impending escalation of care (12, 20). In this respect, Delta MEWS may better reflect evolving instability than a single static value. More broadly, recent work on the duration of hypotension below target mean arterial pressure and on early lactate dynamics in emergency sepsis also supports the importance of time-dependent physiologic change rather than one isolated measurement (21, 22).

### 4.3 Practical workflow implications for emergency nurses and physicians

The nomogram was designed for bedside use from routinely charted variables. Nurses can record M1 within 1 h of observation-room admission, update M2 and SpO_2_ when clinical worsening prompts transfer or urgent reassessment, and estimate the probability of MEWS-based high-alert status using the nomogram. This workflow preserves the clinical logic of the original study: to convert empirical bedside concern into a more structured and visible risk-stratification pAUROCess.

From a workflow perspective, the tool may help nurses prioritize closer observation, earlier physician notification, faster bed allocation in the resuscitation area, and more explicit documentation of escalation triggers. For physicians, the score may support faster synthesis of dynamic physiologic change at handoff and may facilitate decisions about closer monitoring, treatment intensification, and resource allocation. These potential benefits relate mainly to decision support at the time of UTER rather than to pre-transfer screening of the entire emergency observation population.

### 4.4 Circularity, limitations, and future directions

Several limitations should be emphasized. First, because the study included only patients who already underwent UTER, the model cannot be interpreted as a true pre-transfer screening tool for the entire emergency observation population. Second, there is an inherent circularity in using Delta MEWS as a predictor when the modeled endpoint is also defined by M2, because the predictor and endpoint are structurally related. The very large odds ratio for Delta MEWS should therefore be interpreted as reflecting a strong association with a MEWS-based severity threshold rather than an independent external clinical outcome.

Third, the study used retrospective complete-case analysis. Although missingness was clinically estimated to be very low (<1%) because the MEWS and vital-sign data were derived from a structured electronic recording system, the currently available screening logs did not preserve a reliable denominator for all incomplete cases. For this reason, an exact missing-data proportion was not reported, because doing so could create an inaccurate statement. The selection bias introduced by complete-case analysis is therefore likely limited but cannot be fully excluded, and this limitation should be considered when interpreting the findings.

Fourth, the study was retrospective and conducted in three hospitals in China, so generalizability to other institutions and workflows requires caution. Future prospective studies should evaluate whether dynamic markers such as Delta MEWS, serial oxygenation measures, and other time-dependent physiologic indicators can predict independent clinical outcomes before transfer occurs, including ICU admission, intubation, vasopressor use, or short-term mortality (12, 20–22). Such work would clarify the role of this tool as a true early warning instrument beyond stratification at the time of UTER.

Furthermore, AUCPR should be interpreted with caution across cohorts with different prevalences. Unlike AUROC, precision-recall summaries are strongly influenced by the positive-class fraction. Therefore, a numerically lower AUCPR in the external cohort (with much lower prevalence) may still represent substantial enrichment over its low baseline. For this reason, we reported AUCPR alongside the cohort-specific positive-class fraction and emphasize that readers consider both together. Future studies should prespecify the software and integration rule used for AUCPR to ensure comparability.

## 5 Conclusion

In this retrospective multicenter cohort of patients who had already undergone UTER, Delta MEWS combined with SpO2 provided useful bedside stratification of MEWS-based high-alert physiologic status at the time of transfer. The nomogram may assist emergency nurses and physicians in recognizing severe physiologic worsening during transfer-related escalation. The added precision-recall analysis indicates that model performance should be interpreted in relation to cohort-specific class balance rather than AUAUROC alone. However, prospective validation against independent clinical outcomes remains necessary, ideally through a predictive model incorporating dynamically collected hemodynamic variables, hemoglobin, albumin, qSOFA, and related indicators.

## Data Availability

The raw data supporting the conclusions of this article will be made available by the authors, without undue reservation.
